# Factors influencing professionalism during dental training at the University of Chile: perception of students and patients

**DOI:** 10.1186/s12909-023-04329-7

**Published:** 2023-05-17

**Authors:** Marcela Alcota, Juan C. Salinas, Marco Cornejo-Ovalle, Pilar Ruiz de Gauna, Fermín E. González

**Affiliations:** 1 Department of Conservative Dentistry, Faculty of Dentistry, University of Chile, Santiago, 8380492 Chile; 2 Department of Oral Rehabilitation, Faculty of Dentistry, University of Chile, Santiago, 8380492 Chile; 3 Institute for Research in Dental Sciences, Faculty of Dentistry, University of Chile, Santiago, 8380492 Chile; 4 Department of Theory and History of Education, Teacher Training School of Bilbao, University of Basque Country, UPV/EHU, Leioa, Spain

**Keywords:** Dental education, Professionalism, Values, Dental patients, Dental students

## Abstract

**Background:**

The University should be considered a favourable space and agent for the training and transmission of values and attitudes related to professionalism, such as responsibility, teamwork and ethical commitment. In addition, dentistry is a profession with a deep social sense that seeks to solve the oral health problems of the population to improve the quality of life. In this context, our aim was to explore the perception of students and patients about the contribution of the curriculum to the development of professionalism and to identify the factors that strengthen and weaken this perception.

**Methods:**

A qualitative approach was carried out through focus groups and semi-structured interviews with students from the 4th, 5th and 6th year of training and patients treated at the Dental Clinic of our Faculty.

**Results:**

In the opinion of patients and students, the factors that debilitate the training in professionalism are associated with weakened professional values/behaviours in the training, the lack of teacher training of the professors and factors of educational environment. On the contrary, factors strengthening the professionalism are mainly related to hallmark values/ professional behaviours trained in the institution and to the good evaluation by patients. The respondents also perceive the implementation of a new curriculum as a positive factor for the training in professionalism.

**Conclusion:**

The patients and students interviewed believe that the main strength for the training in professionalism in the institution is the development of adaptability for the future professionals to any social context, especially to a vulnerable one, the ability to solve the problems they face and the responsibility towards the patients and their treatment.

## Background

The University should not be considered a neutral place, but a favourable space and agent for the training and transmission of professionalism. Its task is not limited to teaching skills to practice a given profession, in this case Dentistry, but is also to provide a comprehensive training. This implies training students in values and attitudes related to professionalism, such as responsibility, teamwork and ethical commitment [1, 2].

Professionalism in medical/healthcare disciplines has been defined by several authors, such as Masella, who considers that it is the display of high intellectual, technical, and moral qualities and abilities, at the service of patients and community [3]. Another example is the definition proposed by Trathen and Gallagher which is an amended form from the Royal College of Physicians and states that dental professionalism implies a set of values, behaviours and relationships that underpins the trust the public has in dentists [4]. However, all of them are related to the qualities, values and conducts that must be shown by a person in the specific context of a health profession.

At present, the idea of a good professional is not only linked to that of an “expert” competent in a certain area of knowledge, but also to a committed person, morally responsible in the function or activity they carry out. In this respect, the duty of university training is to consider the growth of the individual and the student, with the aim of training professionals of integrity committed to the welfare of society [2].

The Association for Dental Education in Europe (ADEE) has defined the necessary competencies for a dentist, with ethical conduct and social skills being relevant transversal competencies to develop in the future dentists in Europe. In North America, both the American Dental Education Association (ADEA) and the American Dental Association (ADA) have developed chapters incorporating ethical and professional conducts and recommend that all dental schools work on the development of these aspects. [5, 6].

On the other hand, it is important to point out that all the different levels of an institution are involved in the training of professionalism. The institution must promote and explicitly state the professional values and attitudes to which it subscribes and which it intends to train [7].

Dentistry is a profession with a deep social sense that seeks to solve the oral health problems of the population in order to provide wellbeing and improve the quality of life. From that point of view, it is the duty of dental schools to act as training agents and transmitters of professionalism. This must be expressed through the attitude, conduct and social responsibility of those who practice the profession. Working on these aspects should be a constant concern at the core of the dental education provided in dental schools around the world. An innovative curriculum was implemented by our Faculty of Dentistry in 2014, which includes the development of generic skills, highlighting the ethical commitment and social responsibility. This process has considered the following main elements: (i) Courses such as “Psychosocial and anthropological bases of health”, which include the topics of self-criticism in the practice of dentistry and ethics in professional behaviour with patients, teachers and colleagues, all this before starting the clinical subjects; (ii) patient care provided by comprehensive care clinics, which not only emphasise the concept of improving oral health as a whole but will also decrease the risk of considering the patient as a disassociated object; and (iii) early extramural experiences of training in community hospitals, rural villages, etc., with the approach to different socioeconomic and cultural realities. In addition, in the graduate profile of this new curriculum it is stated that “*the professional is expected to act with ethical commitment and social responsibility, growing in his/her profession with proactivity, leadership, creativity, keeping himself/herself updated and improving on a permanent basis”.*

In this scenario, the objective of this investigation was to understand the perception of students and patients about the contribution of the curriculum to the development of professionalism, identifying the factors that strengthen and weaken this perception.

## Methods

### Study design

This study is a qualitative approach and the case study method was used. The qualitative techniques used for data collection were: first, focus groups (n = 20) and later semi-structured interviews (n = 10).

### Study subjects (or informants)

Patients and dental students, at the Faculty of Dentistry of the University of Chile, were invited to express their perceptions about factors influencing professionalism during dental training. In total, thirty subjects were enrolled in the study: 15 students and 15 patients.

### Strategies used in the selection of informants and data collection in focus groups

20 participants, (ten students and ten patients) with the following attributes were selected for focus groups stage.

#### Students

Three students from the 4th year, three students from de 5th year and four students from the 6th (final) year of the career, courses in which clinical care of patients is regularly performed. The students selected had to be more than halfway (50%) through the treatment of their patients.

#### Patients

Ten individuals attended in different undergraduate clinical specialties and at least halfway (50%) through their treatment.

Students and patients received an email inviting them to participate in the focus group and explaining the objectives and their role in the research. Each focus group began with a summary and presentation by the group leader. Subsequently, the participants read and signed the informed consent form for the session to be recorded and transcribed. The dynamics and purposes of the session were explained, which were: (a) To analyse and interpret the meaning of the most important values associated with professionalism in the Faculty according to the results of a previous study [2], (b) to identify the factors or circumstances that enhance and improve professionalism and those that weaken and hinder it, and (c) to analyse the improvements needed in the curriculum and the educational climate to favour the development of professionalism. The notes taken by the researchers during the focus groups and the transcribed audios were analysed and a series of major themes arose, which we called “categories”. The focus groups lasted 1.5 h.

### Identification of key informants and data collection through semi-structured interviews

Ten individuals were additionally selected, five students and five patients, as key informants, different from those who participated in the focus groups. The inclusion criteria for their selection were:

#### Students

Student representatives (delegates) of their respective courses (fourth, fifth and sixth year, courses in which clinical care of patients is performed).

#### Patients

These patients were selected from the complaints book of the clinic, where patients can register their opinions on the care received. Subjects who had problems or dissatisfaction with the dental care provided at the clinic of the Faculty were selected.

The process for conducting the interviews was divided in two stages:

1. Preparation of a guiding script, with questions arising mainly from two complementary processes: (i) The researchers’ previous investigation, literature review and reflection which defined the research objectives; and (ii) the analysis of the results obtained from the field notes and transcription of the focus groups. These processes finally led to the establishment of the major themes to be addressed in the interviews with the key informants [8].

For the validation of the guiding script of the interview, two experts in education, who did not participate in the study, were asked to analyse the questions in relation to their clear comprehension. Then, their corrections and suggestions were introduced. Afterwards, a conversation protocol was designed, where the theme script, a question script, the structure, rhythm and duration of the conversation were established [8]. Table 1 shows the script used in the semi-structured interviews.

The sample size was initially fixed but was open to revision during the course of the investigation if data collection reached the point of information saturation. Information saturation occurs in the course of an investigation when respondents begin to repeat the same concepts and stop contributing new information to the investigation.

**Table 1:** The seven questions included in the guiding script of the semi-structured interviews

QUESTIONS	
1. *How do you perceive training in solidarity at the Faculty and how could we strengthen it?* 2. *Do you think that the career provides the tools for teamwork and work with professional networks from other areas?* 3. *Regarding the participation of the dentist in organisations, do you think it is well developed among professionals and students? (Question addressed only to students).* 4. *In general, do you think that the dental academic of the Faculty of Dentistry at the University of Chile is a good role model for the students?* 5. *In your opinion, what are the values linked to professionalism that can be remarked in the training at the Faculty? On what grounds do you make these comments?* 6. *Do you think that the new curriculum could contribute to the training in values of the students? In what way? What aspects of this curricular innovation would you highlight as relevant in the context of professionalism? (Question addressed only to students).* 7. *What important aspects for a professional graduated from our institution should we work on?*	

2. Invitation sent to the key informants to participate through an email, in which the objective and reasons for the interview and its approximate duration were explained. If they agreed to participate, a personal meeting was arranged with them to clarify the reasons and objectives of the interview and a commitment was made regarding the date, place, guarantee of anonymity, confidentiality of information and request for authorisation to record the interview. They read and signed the informed consent form. The interviews lasted approximately one hour.

### Data analysis

All interviews were transcribed verbatim. A first stage of identification of major themes, categorisation and coding was carried out on paper, with a text analysis of the transcriptions of the audio recordings of the focus groups and semi-structured interviews. Based on the analysis and interpretation of the different texts, the process of definitive categorisation was addressed, which allowed to establish and identify the main thematic nuclei appearing in the data.

In a second stage, the ideas were structured in categories, i.e. the units of information referring to a same topic were grouped conceptually in the thematic nuclei identified.

**Fig. 1:**
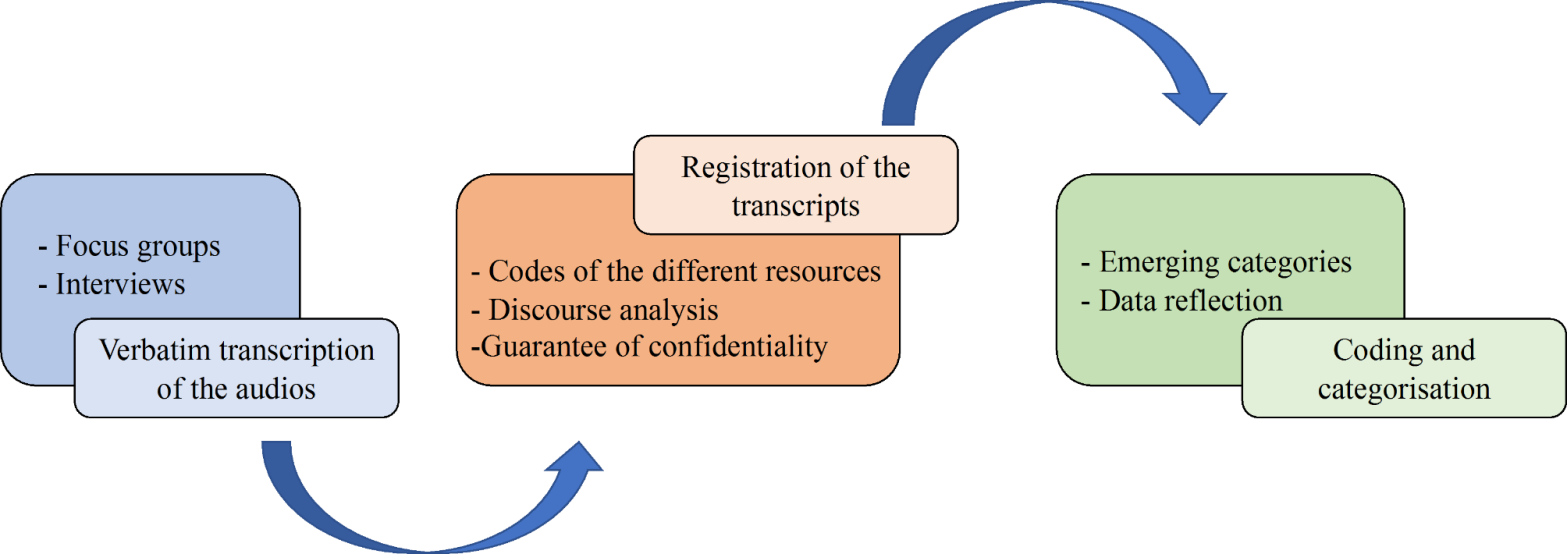
Steps followed to perform the data analysis

Subsequently, this inductive categorisation was transferred to the qualitative analysis software NVivo 11 (QSR International, Melbourne, VIC, Australia), where the transcripts were introduced for coding. At this stage, the discourse analysis was carried out again, focusing on the themes that arose from each of them. Figure 1 shows a scheme of the steps followed in the data analysis.

The information obtained was grouped in a matrix presenting two large dimensions: weaknesses and strengths in the training in professionalism. Each dimension was divided into categories and subcategories. The operational definitions of each category are shown in Table 2.

**Table 2:** Operational definition of the categories

DIMENSIONS	CATEGORIES
**1. WEAKNESSES IN THE TRAINING IN PROFESSIONALISM**	1. WEAKENED PROFESSIONAL VALUES/ATTITUDES This refers to the professional values or attitudes that are explicit in the institutional mission and vision, but not formally addressed in the syllabus and relegated to the sphere of the hidden curriculum.
	2. WEAKNESSES IN THE TRAINING OF THE FACULTY MEMBERS Refers to the need for training of teachers in pedagogy, mainly in relation to the lack of training in methodologies of teaching and evaluation, as well as heterogeneity in the modelling.
	3.WEAKNESSES OF THE EDUCATIONAL ENVIRONMENT Gathers aspects hindering environments of integrity and therefore the teaching-learning of professionalism within the institution.
**2. STRENGTHS IN THE TRAINING IN PROFESSIONALISM**	1. PROFESSIONAL BEHAVIOURS/HALLMARK VALUES Refers to the trained professional values/behaviours related to professionalism that distinguish the institution.
	2. POSITIVE EVALUATION BY THE PATIENTS Gathers the aspects of professionalism rated as positive by patients within the Faculty.
	3. NEW CURRICULUM Includes the aspects of the implementation of the new curriculum considered as positive and that contribute to the training in professionalism.

## Results

Three categories arose in the dimension “weaknesses in the training in professionalism”: weakened professional values/attitudes, weaknesses in teacher training and weaknesses in the educational environment. Table 3 shows the matrix with the final categorical system obtained.

**Table 3:** Categorical system

DIMENSIONS	CATEGORIES	SUBCATEGORIES
	1. WEAKENED PROFESSIONAL VALUES/ATTITUDES	**1. Solidarity** **2. Community work** **3. Teamwork**
**WEAKNESSES IN THE TRAINING IN PROFESSIONALISM**	2. WEAKNESSES IN THE TRAINING OF THE FACULTY MEMBERS	**1. Heterogeneity in the modelling** **2. Lack of training in methodological and evaluation strategies**
	3. WEAKNESSES OF THE EDUCATIONAL ENVIRONMENT	**1. Stress, lack of time and pressure to finish the clinical programmes.** **2. Individualism and competitiveness**
	1. PROFESSIONAL BEHAVIOURS/HALLMARK VALUES	**1. Adaptability to different social contexts** **2. Problem-solving skills** **3. Responsibility and Commitment**
**STRENGTHS IN THE TRAINING IN PROFESSIONALISM**	2. POSITIVE EVALUATION BY THE PATIENTS	**1. Quality and commitment to a work well done.** **2. Empathy and concern for them** **3. Students and faculty members responsibility**
	3. NEW CURRICULUM	**1. Integration and collaboration between teachers and the teaching teams** **2. Early approach to the professional reality**

In relation to the weakened professional values/attitudes in the training in professionalism, solidarity, teamwork and community work were identified. Regarding teacher training, according to the interviewees, the weaknesses are the lack of pedagogical training of teachers and their heterogeneous attitudes and behaviours when facing the same conflict situation. The third category is related to factors in the educational environment that interfere with professionalism within the institution. Among them, stress, competition and individualism were identified (Table 3). Examples of respondents’ opinions in these categories are shown in Table 4.

**Table 4:** Opinions of patients and students related to the dimension “Weaknesses in the training in professionalism”

**Weakened professional values/attitudes**
• *I would like to see a few more students, and let’s say teachers, willing to help, showing solidarity in what they give… I think that somehow all the professions can show some solidarity with the society and the country we live in, and I feel that sometimes health professionals, in general, are a little selfish in this respect, this is what I feel, and I feel that today I cannot pay the bill. Patient* • *This is a university, you don’t come here just to be trained as a professional but also to be trained as a person, I think that there is a lack of space for diffusion, dialogue, giving ideas, solving problems, and here, I don’t know …I think that those who participate in institutions, organisations must be … 5%? It’s very little, that is, almost nothing. Student* • *We have always been told to relate to each other, to work as a team, interdisciplinary and all that, but I think that little is done to make us relate to each other. Student*
**Weaknesses in the training of the faculty members**
• *There are no teachers here, here the teachers are specialists who come and teach you their specialty, but they don’t have the pedagogical or teaching part they should have. Student* • *It affects when the teacher starts to be unpleasant and has a not very professional behaviour, because in the end you somehow try to imitate the teacher, or follow him, these behaviours are stuck in you. Student* • *Once I had a patient with cancer and he/she had to have an operation … he/she started to tell me his/her things because he/she wanted a dental treatment and the teacher said in front of the patient “don’t get involved in this mess, this patient is going to die any minute and you are going to be left halfway through”. Student* • *I think that the fact to scold or correct a student should be made in a more pedagogical manner, not as angrily as I once witnessed. Patient* • *It struck me today when the student was much more sympathetic towards my problem than the teacher, who said:” just close it”. The student took care of my tooth, she did it with affection. I feel that this is very important and hopefully she won’t lose it in the future when she becomes a professional. Patient*
**Weaknesses of the educational environment**
• *This is a very controversial topic because from the fourth year onwards, people show how they really are, because people who were good people, or good students, become totally different people in order to finish their treatment, being sure that they can do anything to finish their programme and they don’t give a damn about the rest. Student* • *The students have a different view of what they are doing. Here they are doing it for their career, here they are doing it for their course, here they are doing it so that their job is considered well done, in order to pass the course, this is their goal, so in this regard their behaviour is obvious. When they reach their goal, they have to live, and within that living is the fact that they dehumanize themselves. Patient*

Regarding the dimension “strengths in the training in professionalism”, the data were grouped into three categories: professional behaviours/hallmark values, positive evaluation by the patients attended in the institution and implementation of a new curriculum of the career (Table 3).

In the category “professional behaviours/hallmark values”, adaptability, problem-solving skills and responsibility in clinical patient care were identified as the main strengths of the training provided by the institution to its students. In the category “positive evaluation by the patients attended at the institution”, the most mentioned attributes related to professionalism are quality of work, commitment, empathy, concern and responsibility. Finally, the third category of this dimension “implementation of a new curriculum” is a strength in the training in professionalism, according to the opinion of the interviewees, since it has allowed the integration and collaboration between dental disciplines and has also led to an early approach to the professional reality, not only in the last years of the career as before (Table 3). Some of the opinions of the interviewees in this category are shown in Table 5.

**Table 5:** Opinions of patients and students related to the dimension “Strengths in the training in professionalism”

**Professional behaviours/hallmark values**
• *The number of graduates who work in the public service is important, they get involved because they want to be dentist in rural areas, most of them are interested in the public service. Student* • *A professional from the University of Chile knows how to work it out with the tools available in order to treat a patient properly and to help him/her, I think that in a health centre, let’s say, in a very rural area, he/she will be able to work things out to attend his/her patient. Student* • *I think that it is important to know how to face the problems and try to find a solution with the resources available, and maybe all these difficulties we have make us strong in this aspect. Student* • *I think it is like the responsibility, reliability they show towards their patients, the responsibility towards what they assume. Patient*
**Positive evaluation by the patients**
• *The students take it very seriously, very responsibly and make great efforts, and it struck me because it is not easy to find it … the teacher is attentive, he/she is looking around and knows where to check again soon, so you can feel this atmosphere of tremendous professionalism that you don’t find anywhere else… Patient* • *I have been attended very well and they also make sure that everything fits well; the same goes for me as a senior citizen, if I have a problem, if I feel bad at any time, they are always extremely concerned, teacher and student, so I am very happy… Patient* • *In these three years, I have seen that student and teacher care is directed towards doing things right, doing things in an excellent way, in other words, it has to be done better than just well… Patient* • *They (the students) have been extremely sympathetic, I don’t know, what can I tell you, I’ve been through tough family stuff, they heard about it and came to ask me… so this … it fills me … because I totally trust these kids, totally … Patient* • *I think they are the best, I am “pro-University of Chile” and for me the professionals graduated from this University are really the best, without a doubt. Now, about their values … the commitment, the efficiency … I have the best opinion of the professional from the University of Chile … Patient*
**New curriculum**
• *The training is comprehensive and so they will not ask for programmes but rather for comprehensive discharges, where you understand that you are treating patients, not teeth, so this is where the more professional speech should be directed, that is the commitment of the student towards the patients, not towards his/her study programme, I think that in this sense, it is positive. Student* • *I think it is very good that instead of focusing on the number of actions, it focuses on a patient in a comprehensive way. Student*

## Discussion

The objective of this study was to understand the perception of students and patients regarding the training in professionalism in the Faculty of Dentistry at the University of Chile. To this end, by means of focus groups and semi-structured interviews, we identified the professional values/behaviours students and patients consider to be present in the training provided by our faculty, and the professional factors they consider to be weakened. In addition, we investigated which educational factors within the faculty weaken or strengthen the teaching and learning of professionalism.

Among the weakened professional values/behaviours, i.e. those that are not being taught during professional training or not being reflected in the behaviour and conduct of the students and/or other members of the institution, we find: solidarity, participation in the community and teamwork.

Regarding solidarity, patients perceive that professional training is not aimed towards teaching solidarity, which is expressed when health professionals, after graduating, do not take or take few actions for the community, where this value is mainly reflected.

There is evidence in the literature that part of the dental students are individualist and competitive, unconcerned about others and their environment; that is, they lack solidarity. These personality traits might even be the reason why some of them choose to study dentistry [9–11]. In this regard, it is also noted that often the attitude of the professors contributes to fostering these attitudes among students, by recognizing and praising those who get a high grade in a course or who manage to finish the clinical programmes first and not giving equal recognition to the students who show solidarity towards their patients and/or classmates [12–14].

In our study, students think that solidarity is not present in the training, either in the curriculum or in the teaching practice. Interestingly, students perceive that this value is weakened mainly when they start the clinical care, since they have to complete clinical actions and programmes in the different subjects. Therefore, they have to make sure they have enough patients to complete the programmes. On the one hand, this creates a competition among the students to obtain patients, and on the other hand, makes that the patient is only seen as a programme to be completed. This situation has also been described by different authors [12, 15, 16].

Regarding the participation in the community, several publications have reported that there is no emphasis on the social role and responsibility of a dentist as an individual inserted in the society in the curricula of faculties of dentistry in Latin America and the world. Therefore, a disconnection exists between the training given in educational institutions and the needs of the community. For this reason, many dental schools are making a major effort to include courses and credits in their curricula, aimed at developing this ability in students through early approaches to community service work [17, 18].

In this work, the students think that, even with the best intentions and considering the importance of the link with the community, the syllabus does not include spaces to generate activities related to the participation in the community. The Faculty has an Outreach Office that provides a great support for community work. However, this is mainly an extracurricular activity for the students and in many cases their participation is limited because of the academic load and lack of time. The patients attended at the Faculty state that they realize that the students do not have time for community work and only want to rest, given the pressure and fatigue produced by the career. Besides, they suggest that it would be important to give more information to the community (in the municipality, neighbourhood councils, health centres etc.,) about the actions and cares provided by the Faculty, which they value and understand as a service to the community. They point out that people are not informed and unaware of the different activities and services provided by the institution. In this context, research shows the need for a deeper integration of the clinical actions with the outreach activities.

With regard to teamwork, the evidence shows the need to promote a greater interaction and integration among students with other disciplines in the health area, in order to change the concept of individual and solitary work in the dental chair [16–18]. A study carried out in 2011 in the Faculty of Dentistry of the University of Chile described that, according to the opinion of students and academics, teamwork was one of the least trained and least achieved competences in the institution [19].

This study aimed to study teamwork in depth, not only from the perspective of multidisciplinary work, but also from the training of future dentists as professionals who participate in professional and guild organisations. These aspects arose from the focus groups.

The opinion of students regarding the multidisciplinary nature of the profession is that it only exists among the different dental specialties, even though they recognise that many dentists find it difficult to refer patients to other colleagues from different specialties. In relation to the other health professions, the interaction between disciplines is much less frequent. According to the opinion of the interviewees, this may be explained by two factors: the isolation of the Faculty or its little or limited contact with other health careers, and the lack of comprehensive training, where the patient is only seen as an individual suffering from an oral health problem dissociated from other diseases they may have.

The participation in organisations is much weaker. The training does not promote this aspect, and if it happens, it is due to the proper characteristics of the person who has the sensitivity and interest in working in this type of organisation. The respondents, patients and students, consider it very important to receive training and incentives to participate in these organisations, in view of the need to have some influence in the oral health policies of the country.

The second category identified in the dimension “weaknesses in the training in professionalism” was “weaknesses in teacher training”. Several studies point out that, for a good teaching-learning process, a relevant aspect is the training of teachers in university pedagogy. This way, they can teach and evaluate using a variety of strategies, not only specific professional skills but also the behaviours and attitudes of the students. This is especially important when they are working in a challenging and stressful environment, such as the clinical care of patients [20–22].

When analysing teacher training according to the discourse of the participants, two aspects in which training is weakened become evident: (a) The heterogeneity of teachers in terms of being role models for the students, and (b) the lack of improvement in teaching methodologies and assessment tools.

In education, role modelling states that teachers are examples of professionalism, behaviour and attitude. On the contrary, some teachers may de-professionalise the students [7]. It is important to mention that both groups interviewed acknowledge the fact that teachers are well-trained in the scientific and technical aspects of the profession. However, this contrasts with the perception of students, and sometimes patients, regarding the attitude of some teachers who, for example, leave the students alone in the clinic when they are attending patients or who strongly reprimand them in front of the patients. Besides, both students and patients report a lack of appropriate training in university pedagogy of the teachers. This would provide them with knowledge of active methodological strategies and adequate forms of evaluation of the teaching-learning process, in both the technical aspects of the profession and the training in professional values/behaviours, which is in concordance with various publications in dental education [20–22].

With respect to the third category, “weaknesses of the educational environment”, the highly competitive individualistic environment existing in the Faculties of Dentistry does not favour the personal and human development of the students [15]. Moreover, many studies mention stress, lack of time, and exhaustion as factors that do not help to create an environment of integrity, favourable to generate behaviours linked to professionalism [10, 19, 23–26]. From this point of view, the groups interviewed recognise that pressure and stress to pass the courses lead sometimes students to carry out dishonest and unprofessional actions. Although this is not a generalized situation, they think that it weakens the perception and environment of professionalism in the educational context.

Regarding the “professional behaviours/hallmark values” category, associated with the dimension “strengths in the training in professionalism”, the respondents remarked that the capacity to adapt and behave professionally in any social context is something developed in the training. In other words, at the University of Chile, a student knows how to behave in all respects, i.e. in their attitude towards the patient and with the appropriate management of knowledge and infrastructure in both a vulnerable institution with scarce resources and in one with optimal infrastructure and technology.

Being a professional capable of adapting may be explained by the fact that the clinical care at the Faculty of Dentistry is provided in a neighbourhood with vulnerable socioeconomic context. Therefore, as it has been shown in different studies, as students experience social inequalities in their training, they become more prepared to confront them and work for them [27–29].

Another hallmark-value of the student graduated from the University of Chile is his/her capacity to solve most of the problems that arise in dental practice and work effectively with the tools available. This may be one of the most important competences to be developed in future professionals. In the context of the Faculty, we believe, like many interviewees, that it is obtained not only through the good scientific training but mostly through the extramural training where they carry out their practice once they graduate. This extramural training takes place in contexts of vulnerability associated with public healthcare in the country. Examples of the opinion of the interviewees in relation to the professional behaviours/hallmark values are shown in Table 4.

Another relevant aspect of this study was to address the perception of the patients on their dental care and treatment at the Faculty of Dentistry from the point of view of professionalism. It is important to mention that there are two main groups of patients who come to the dental clinic of the Faculty. On the one hand, patients who have frequently been treated at our clinic in several disciplines and/or subjects and who recommend the Faculty to their family and friends. On the other hand, patients who live in the area or who have been informed that the institution provides treatment at a lower cost.

When interviewed about the behaviours/attitudes associated with professionalism in the training at the Faculty, most of the patients remarked that the quality of the work performed and the commitment and dedication in doing this work well were the main reason why they came to the institution. Besides, they highlighted as professional values the concern towards them as individuals, the welcoming attitude, the empathy, and the fact to be treated very well and with responsibility, from both students and teachers. Examples of the discourse of the patients in relation to the training in professionalism in the institution are shown in Table 5.

It is important to remark that from the interviews with the patients, we observed that they create very important and deep affective and human bonds with the students, which has also been described previously [30]. This mainly happens in the longer treatments in the specialties of removable prostheses, comprehensive clinic, periodontics and complete prostheses, where the patient is an adult or older adult, which creates a paternalistic vision of admiration and affection towards those “kids” trained to become excellent professionals. In addition, as observed by Lowe et al., the altruistic attitude of the patients stands out [30].

The patients also stated that the relationship between them and the institution has to be mutually supportive, in the sense that they go to the clinic knowing that those who give the care are students and, therefore, there is a possibility of mistakes during this process. From this point of view, they highlight the fact that the care should be lower in cost, as the fees of the Faculty are not so different from other private dental clinics in the area (vulnerable area). In this regard, it is important to note that several faculties around the world offer free dental care or at minimal cost [30].

Finally, when analysing the category “implementation of a new curriculum”, which began in 2014, within the dimension of “strengths in the training in professionalism”, it is important to note that in its curricular innovation the Faculty of Dentistry of the University of Chile follows the guidelines of a hybrid curriculum. Because it is the most traditional and oldest Dental School in the country, it was considered very difficult to change the departmental and disciplinary structure, as it happened in other European and North American universities when conducting this type of innovation [31].

The current vision is for the disciplines to work in a comprehensive and articulated manner so that, on the one hand, the teaching-learning process becomes more holistic and with criteria involving agreements, collaboration and teamwork for the best care and solution to the health problems of the patients. On the other hand, both the methodologies and instruments of evaluation need to be consistent with the learning objectives. In addition to the practice in comprehensive clinics, the new curriculum considered, at least in its design phase, exposing students to situations closer to the professional reality from an early stage of their training [17, 18, 32].

The interviewees express the hope that this innovation will indeed contribute to the training in professionalism. In this sense, they value positively the interaction of the different disciplines that is already beginning to take place, which has resulted in teachers meeting and coordinating with each other, which hardly happened in the old curriculum. Another positive aspect is the comprehensive nature of the clinics, which means that the actions are no longer conducted by discipline or specialty but by the comprehensive treatment of the patient. In this sense, and in agreement with the scientific literature, this also helps to see the patient not as a programme to be fulfilled or a number of actions, but as an individual, whose oral health is being restored and who is being helped to remain healthy [10, 19]. Examples of the interviewees’ opinions regarding the implementation of the new curriculum and professionalism can be seen in Table 5.

## Conclusions

According to the opinion of the patients and students interviewed, the ability to adapt to any social context, especially vulnerability, the ability to solve problems and the responsibility towards the patients and their treatment are the values that distinguish the training in professionalism at the Faculty of Dentistry of the University of Chile. On the other hand, the professional behaviours/values important for the different interviewees but weakened in the training are solidarity, participation in the community and teamwork.

Teacher modelling towards the students is very heterogeneous, that is, many teachers are indeed role models and examples of virtue, while some are quite the opposite. In this sense, it is vital to promote a reflective attitude towards their teaching practices, both in the teaching of their discipline and in the behaviours and attitudes associated with professionalism. In addition, these practices should also be based on innovation and on the evaluation of processes and results. Along with this, a joint reflection with the teachers on the training actions necessary to progress in the improvement of aspects related to professionalism is important, considering the opinions of each teacher, the teaching teams and the institution.

On the other hand, a large majority of the groups interviewed thinks that the highly competitive, individualist and stressful climate hinders the training and practice of professionalism. In this context, the need to transform the institutional culture, in terms of the relationships of its members, to a more humanised and people-centred environment is highlighted.

Finally, the implementation of a new curriculum aimed at achieving clinical competences and comprehensive patient care is another factor that the interviewees believe will strengthen professionalism. Indeed, it encourages the student to see the patient as a person to be helped to solve their oral health problems and not as an object or programme to be fulfilled. With this curricular innovation, we have begun to move from a teaching culture based on discipline and individuality to an interdisciplinary organisation based on collaboration in the teaching-learning process, from a curriculum based on a sum of unconnected subjects, to a comprehensive approach where disciplines converge, creating new courses and teaching teams. The establishment of multidisciplinary teaching teams that try to collaborate with each other is another positive aspect. We believe that this new way of working should improve the educational climate and, therefore, the training in professionalism.

Although the conclusions of this research are based on one case study, we believe that the object of study of this work, as well as its analytical approach, can be extremely useful and provide valuable information in other educational contexts in Latin America and the rest of the world.

## Future perspectives

This work suggests the need for future research in this line, but delving into other aspects:

It is relevant to develop the process of identity construction and teaching culture of the faculty members of the Faculty of Dentistry at the University of Chile, especially at a time of a complex curricular innovation. It is also important to investigate what the academics understand about being a teacher at the University and in the Faculty, what their motivations are, what their professional values should be, and what they understand by teaching and research.

Another aspect to explore in depth is the perception of the patients regarding dental care at the Faculty, in relation to the most important personal and behavioural attitudes of those who give care. For example, whether or not physical appearance, such as clothes or the use of uniform, jewellery or tattoos affect the perception that the patients have of the professional.

This work was conducted before the health crisis generated by the COVID-19 pandemic. Therefore, it would be relevant to explore how the pandemic has changed professional behaviour in dental care and the training in professionalism, and from the point of view of each of the groups of our educational community.

Finally, it is important to note that a curricular adjustment of the career is planned for 2024. Indeed, progress will be made in aspects related to professionalism in real contexts during the last years of the training. Thus it will be addressed transversally in the six years of the career and not only in the initial years.

In addition, we will work with clinical teachers on improving their teaching and evaluation strategies in seminars and courses informing them on the importance of teaching by modeling and promoting probity in clinical-practice teaching.

## Data Availability

The data that support the findings of this study are available on request from the corresponding author. The data are not publicly available due to privacy or ethical restrictions.

## List of Abbreviations

ADA: American Dental Association.
ADEA: American Dental Education Association.
ADEE: Association for Dental Education in Europe.
